# Multitrajectories of Frailty and Depression With Cognitive Function: Findings From the Health and Retirement Longitudinal Study

**DOI:** 10.1002/jcsm.13795

**Published:** 2025-04-06

**Authors:** Chengxiang Hu, Xiaoyue Sun, Zhirong Li, Yue He, Beibei Han, Zibo Wu, Siyu Liu, Lina Jin

**Affiliations:** ^1^ Department of Epidemiology and Biostatistics, School of Public Health Jilin University Changchun Jilin Province China; ^2^ State Key Laboratory for Diagnosis and Treatment of Severe Zoonotic Infectious Diseases Key Laboratory for Zoonosis Research of the Ministry of Education Changchun Jilin Province China

**Keywords:** cognitive decline, cohort study, depression, frailty, multitrajectory

## Abstract

**Background:**

Little is known about the joint associations between trajectories of frailty and depression with cognitive function. This study aims to explore the multitrajectories of frailty and depression and their joint impact on cognition.

**Methods:**

A total of 8600 participants from the Health and Retirement Study (HRS) (1996–2018) were analysed using a group‐based trajectory model for 10‐year multitrajectories. Participants were classified into five groups based on their trajectories. Multivariable linear mixed models and Cox proportional hazards models were utilized.

**Results:**

Compared with Group 1 (stable robust and nondepressed), Groups 2 (‘worsening prefrailty without depression,’ *β* = −0.022 SD/year), 3 (‘stable prefrailty with escalating depressive symptoms,’ *β* = −0.016 SD/year), 4 (‘increasing frailty alongside worsening depressive symptoms,’ *β* = −0.034 SD/year) and 5 (‘high and escalating frailty with persistent depression,’ *β* = −0.055 SD/year) exhibited accelerated cognitive decline. Dementia risk was significantly higher in G2 (HR = 1.26, 95% CI: 1.08–1.48), G3 (HR = 1.54, 95% CI: 1.31–1.80), G4 (HR = 1.81, 95% CI: 1.54–2.14) and G5 (HR = 1.86, 95% CI: 1.48–2.33) compared with G1.

**Conclusions:**

Worsening frailty and depression accelerate cognitive decline and risk of dementia, underscoring the need to address both conditions to mitigate cognition.

## 1 Introduction

The number of people with dementia is increasing, which is projected to be doubled by 2050 with the global aging population [1, 2]. Dementia not only severely impacts patients' quality of life but also burdens families and healthcare systems [3]. However, no effective treatment for dementia is reported currently [2]. Cognitive decline is a fundamental characteristic of dementia and can be considered a progression from cognitive health to dementia [4]. Therefore, investigating new risk factors that predict cognitive decline is an effective approach for understanding and preventing dementia.

Research has shown that decreasing modifiable risk factor levels is associated with improved cognition and a reduced risk of dementia in mid to late life [5]. For example, healthy lifestyle (such as regular exercise) and reducing air pollution are linked to lower dementia risk [6, 7]. Frailty and depression, as modifiable cognitive risk factors, have been extensively studied for their associations with cognitive decline and dementia [8, 9]. As a common mental health disorder, depression has been identified as a risk factor for cognitive decline [2, 10]. In addition, frailty is characterized by reduced functioning of multiple physiological systems and increased vulnerability to stressors [11]. Several studies have established a relationship between frailty and cognitive impairment [12, 13], and some evidence shows that people with multiple chronic and serious diseases are at higher risk of dementia, especially those with diseases begin in midlife [5].

The aforementioned studies explored the independent effects of frailty and depression on cognitive function. However, recent research suggests a bidirectional relationship between frailty and depression, indicating that frailty and depression may have a combined impact on cognitive function [14, 15]. Moreover, much of the existing research on the effects of depression and frailty on cognition has been conducted at a single or two time points [16, 17], whereas both frailty and depression are dynamic and susceptible to change over time [2, 18].

Therefore, we utilized data from the Health and Retirement Study (HRS), which features long‐term and repeated assessments, to investigate the longitudinal trajectories of frailty and depression. This study aims to explore the combined effects of the long‐term changes in frailty and depression on cognitive decline and dementia.

## 2 Materials and Methods

### 2.1 Study Design and Population

We used longitudinal data from the existing community‐based HRS. HRS uses random sampling to select the study population that targets a nationally representative sample of community‐dwelling adults in the United States aged 50 years and older. HRS study began in 1992, with follow‐up every 2 years, and data are available for 14 sessions through 2018. The HRS was approved by the University of Michigan Institutional Review Board and the National Institute on Aging (HUM00061128), and written informed consent was obtained from all participants. The data collection approach for this cohort has been described in previous related studies [19].

We used data from six cycles to fit the trajectory. Wave 3 of the HRS (1996) was considered the baseline for our study. People who participated in Wave 3 were involved. Participants with (1) missing > 20% items of frailty index in more than three waves, (2) missing complete depression questionnaires in more than three waves, (3) loss to follow up (Waves 8–14) and (4) dementia were then excluded. This study finally included 8600 subjects; see details in Figure S1.

### 2.2 Assessment of Frailty

Frailty was assessed using a 61‐item Frailty Index (FI) based on a health deficit accumulation model (Table S1) [11, 20]. The FI was constructed following a standardized 10‐step procedure, including variable selection, handling missing data, recoding responses (0 = no deficit, 1 = deficit), screening for age associations and variable correlations and calculating scores as the proportion of deficits present. [20]. FI scores ranged from 0 to 1 and were multiplied by 10 for clarity. Frailty status was categorized as robust (FI ≤ 1), prefrailty (1 < FI ≤ 2) and frailty (FI > 2), with higher scores indicating greater frailty. The details of FI are shown in Figures S2–S3 and Supplementary description.

### 2.3 Assessment of Depression

Measures of depressive symptoms were based on the eight items of the Center for Epidemiologic Studies Depression Scale (CES‐D), which is divided into six negative and two positive items, with the positive items reverse‐coded. A total CES‐D score was obtained based on the participant's responses, ranging from 0 to 8, with higher scores representing greater levels of depressive symptoms. As in a previous study [21], participants with a total CES‐D score equal to or higher than 3 were classified as depressed.

### 2.4 Assessment of Cognitive Decline and Dementia

Cognitive decline assessment covered three cognitive domains, including memory, executive functioning and orientation [22], and was conducted during Waves 8 through 14 of the HRS. Memory is tested by recalling 10 unrelated words, including immediate and delayed recall, on a scale of 0–20, with one point awarded for each word recalled at a time. Orientation ability, with a score range of 0–4, was assessed by four questions about the year, month, day of the month and day of the week. Executive functioning scores range from 0 to 7 and are assessed through a countdown test (0–2 points) and a sequential 7‐point test (0–5 points), with the total score obtained by adding the scores from both tests.

Similar to previous studies [22], cognitive *Z*‐scores were generated for the purpose of assessing overall cognitive decline. The *Z*‐scores were all generated in two steps. Step 1: Each domain *Z*‐score was generated from a standardized baseline. This was calculated by subtracting the mean from the test score and dividing by the standard deviation (SD) of the baseline domain scores. Step 2: The mean scores of the three domains are renormalized to the baseline, and an overall *Z*‐score for each wave is calculated.

Moreover, dementia was defined as a cognitive summary score of 6 or below on a standardized 0–27 scale, which included immediate and delayed recall, serial 7s and backward counting [23].

### 2.5 Assessment of Trajectory Group

Data from Waves 3–8 were used. Group‐based trajectory modelling (GBTM), which employs finite mixture model to approximate unknown distributions of trajectories across a study population, was used to evaluate the long‐term multitrajectories of frailty and depression over a 10‐year period (at least three biennial assessments) [24, 25].

First, we constructed models with two to six trajectory groups. The appropriate number of classes was determined using the Bayesian Information Criterion (BIC), with a lower absolute value indicating a better model fit. After determining the number of classes, we tested different functional forms to identify which shapes best fit the data. We used high‐order polynomials to determine subgroup shapes; if the high‐order terms were not significant, we applied reduced‐order fits. Finally, based on criteria such as the lowest BIC values, an average posterior probability ≥ 70% and a minimum sample size of ≥ 5% for each class [26], we identified five groups. Specially, Group 1 (G1) is defined as ‘stable robust and nondepressed’, Group 2 (G2) as ‘worsening prefrailty without depression’, Group 3 (G3) as ‘stable prefrailty with escalating depressive symptoms’, Group 4 (G4) as ‘increasing frailty alongside worsening depressive symptoms’ and Group 5 (G5) as ‘high and escalating frailty with persistent depression’. Details of the model selection process were provided in the Supplemental Methods and Tables S2–S5.

### 2.6 Covariates

We included the following baseline covariates as potential confounders [22]. Self‐reported socio‐demographic characteristics included age (years), sex (male or female), race and ethnicity (white; other), wealth (in tertile), living alone status (yes; no), marital status (married; other), educational level (below high school; high school; above high school), smoking status (current; former; never) and drinking once or more per week (yes; no).

### 2.7 Statistical Analysis

The baseline characteristics of participants were summarized by trajectory groups, with categorical variables reported as counts (%) and continuous variables reported as means (SD) or medians (IQR). Differences in trajectory groups were analysed using chi‐square tests for categorical variables and ANOVA or Kruskal–Wallis rank‐sum tests for continuous variables.

In line with previous research, linear mixed models were used to handle multiple repeated measurements of continuous outcome variables [27]. To explore the association between trajectory groups and the cognitive decline as well as the rate of annual cognitive decline (SD per year) during follow‐up, linear mixed models were employed to estimate regression coefficients (*β*) and 95% confidence intervals (95% CIs), with ‘G1’ serving as the reference category. The models included an interaction term (trajectory groups × follow‐up duration) to assess the rate of cognitive decline (SD/year), with random intercepts and slopes to account for individual differences.

Cox proportional hazards models were used to evaluate the association between trajectory groups and dementia risk, estimating HRs and 95% CIs. Person‐years were calculated from baseline questionnaire completion until dementia diagnosis, death or end of follow‐up, whichever occurred first. The proportional hazards assumption was tested using Schoenfeld residuals.

Model 1 was adjusted for baseline age and sex. Model 2 was further adjusted for race, marital status, educational level, wealth (in tertile), living alone, married status, smoking status and drinking once or more per week.

To assess the robustness of our findings, we conducted sensitivity analyses using composite cognitive scores derived from the summation of three domain‐specific measures.

The ‘traj’ package in Stata 17.0. was used to fit multitrajectories. Statistical analyses were based on R statistical software (version 4.3.1; R Core Team). Linear mixed models were performed using the R package ‘lme4’. A two‐sided *p* < 0.05 was considered statistically significant.

## 3 Results

### 3.1 Basic Characteristics of Participants

**FIGURE 1:**
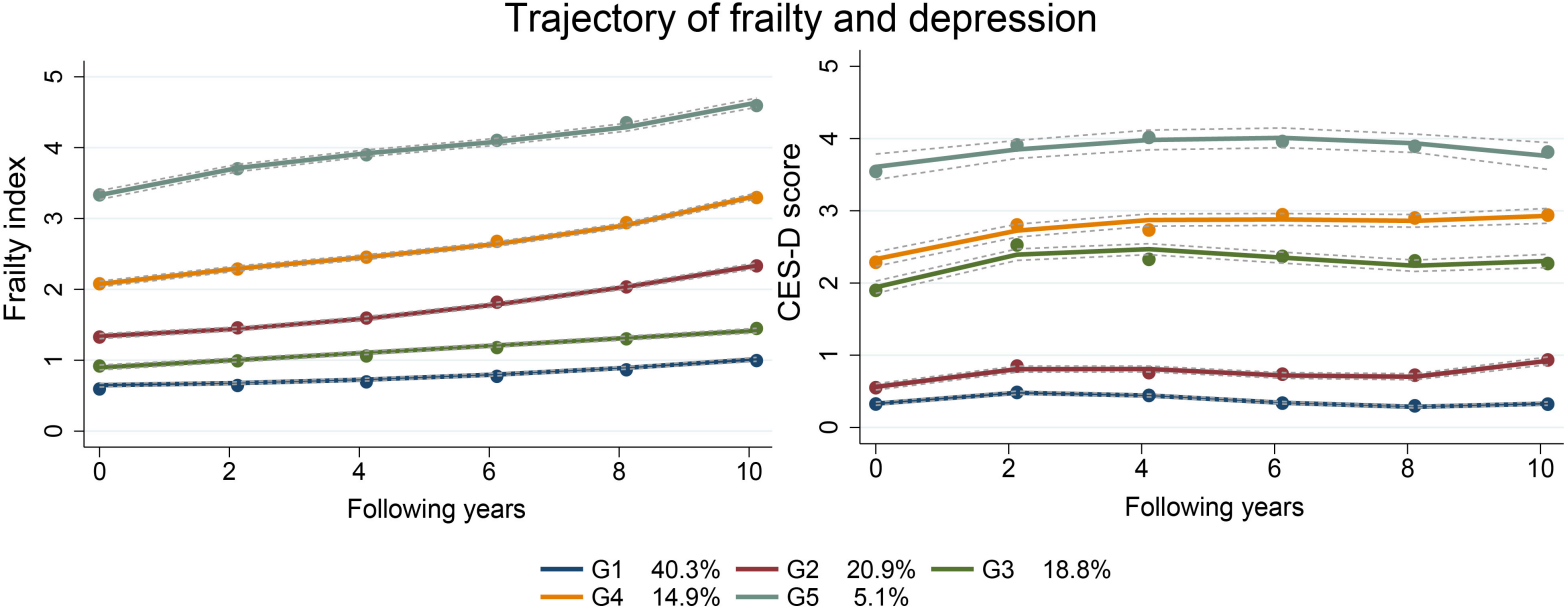
Multitrajectories of frailty and depression from Wave 3 to Wave 8 (1996–2006). G1: stable robust and nondepressed; G2: worsening prefrailty without depression; G3: stable prefrailty with escalating depressive symptoms; G4: increasing frailty alongside worsening depressive symptoms; G5: high and escalating frailty with persistent depression.

Trajectories were labelled as G1 (*n* = 3463, 40.3%), G2 (*n* = 1801, 20.9%), G3 (*n* = 1617, 18.8%), G4 (*n* = 1285, 14.9%) and G5 (*n* = 434, 5.1%). See details in Figure 1. Five groups differed significantly in the composition of each covariate (Table 1).

**TABLE 1:** Baseline characteristics of multitrajectory groups of frailty and depression.

Variables	Overall	G1	G2	G3	G4	G5	*p*
Number	8600	3463	1801	1617	1285	434	
Age, years, mean (SD)	62.29 (7.64)	60.69 (6.52)	64.48 (8.14)	61.36 (7.28)	64.32 (8.53)	63.38 (8.50)	< 0.001
Sex, %							< 0.001
Male, %	3217 (37.4)	1584 (45.7)	703 (39.0)	499 (30.9)	350 (27.2)	81 (18.7)	
Female, %	5383 (62.6)	1879 (54.3)	1098 (61.0)	1118 (69.1)	935 (72.8)	353 (81.3)	
Race, %							< 0.001
White	7224 (84.0)	3064 (88.5)	1519 (84.3)	1302 (80.5)	1034 (80.5)	305 (70.3)	
Others	1376 (16.0)	399 (11.5)	282 (15.7)	315 (19.5)	251 (19.5)	129 (29.7)	
Educational level, %							< 0.001
Below high school	1987 (23.1)	463 (13.4)	395 (21.9)	440 (27.2)	471 (36.7)	218 (50.2)	
High school	5025 (58.4)	2076 (59.9)	1083 (60.1)	962 (59.5)	712 (55.4)	192 (44.2)	
Above high school	1588 (18.5)	924 (26.7)	323 (17.9)	215 (13.3)	102 (7.9)	24 (5.5)	
Wealth (in tertile), %							< 0.001
1st (the poorest)	2885 (33.6)	763 (22.0)	562 (31.2)	626 (38.7)	645 (50.2)	289 (66.6)	
2nd	2848 (33.1)	1204 (34.8)	639 (35.5)	534 (33.0)	372 (28.9)	99 (22.8)	
3rd (the richest)	2866 (33.3)	1496 (43.2)	600 (33.3)	456 (28.2)	268 (20.9)	46 (10.6)	
Married status							< 0.001
Married	5107 (61.6)	2472 (74.5)	1087 (62.1)	819 (52.6)	578 (46.5)	151 (35.6)	
Other	3181 (38.4)	844 (25.5)	663 (37.9)	737 (47.4)	664 (53.5)	273 (64.4)	
Living alone, %							< 0.001
Yes	1340 (15.7)	383 (11.1)	305 (17.0)	288 (17.9)	265 (20.8)	99 (23.0)	
No	7220 (84.3)	3069 (88.9)	1488 (83.0)	1320 (82.1)	1011 (79.2)	332 (77.0)	
Smoking status, %							< 0.001
Current	3677 (44.3)	1512 (45.1)	787 (45.0)	719 (46.4)	483 (39.0)	176 (42.5)	
Former	3294 (39.7)	1358 (40.5)	751 (42.9)	535 (34.6)	499 (40.3)	151 (36.5)	
Never	1334 (16.1)	484 (14.4)	212 (12.1)	294 (19.0)	257 (20.7)	87 (21.0)	
Drinking once or more per week, (%)							< 0.001
Yes	5767 (69.7)	1947 (58.8)	1279 (73.2)	1111 (71.5)	1043 (84.0)	387 (91.3)	
No	2512 (30.3)	1366 (41.2)	468 (26.8)	443 (28.5)	198 (16.0)	37 (8.7)	
Frailty index, mean (SD)	1.17 (0.91)	0.59 (0.36)	1.35 (0.59)	0.92 (0.51)	2.08 (0.79)	3.34 (1.02)	< 0.001
CES‐D score, mean (SD)	1.13 (1.76)	0.33 (0.72)	0.55 (0.92)	1.93 (1.97)	2.28 (2.21)	3.57 (2.46)	< 0.001

Abbreviations: BMI, body measure index; CES‐D: Center for Epidemiologic Studies Depression Scale; G1: stable robust and nondepressed; G2: worsening prefrailty without depression; G3: stable prefrailty with escalating depressive symptoms; G4: increasing frailty alongside worsening depressive symptoms; G5: high and escalating frailty with persistent depression; SD: standard deviation.

Table 1 presents the basic characteristics of participants grouped by multitrajectory groups of frailty and depression. Of the 5306 subjects, the mean age was 62.29 years, the mean frailty index was 1.17, and the mean CES‐D score was 1.13. A total of 5383 (62.6%) of the participants were female, 7224 (84.0%) were white, more than half of the participants had a high school education (58.4%), 5107 (61.6%) were married, 5767 (69.7%) subjects drank alcohol one or more times per week, and 3677 (44.3%) were current smokers. Only 1340 (15.7%) subjects lived alone.

### 3.2 Multitrajectories of Frailty and Depression With Cognitive Decline

**FIGURE 2:**
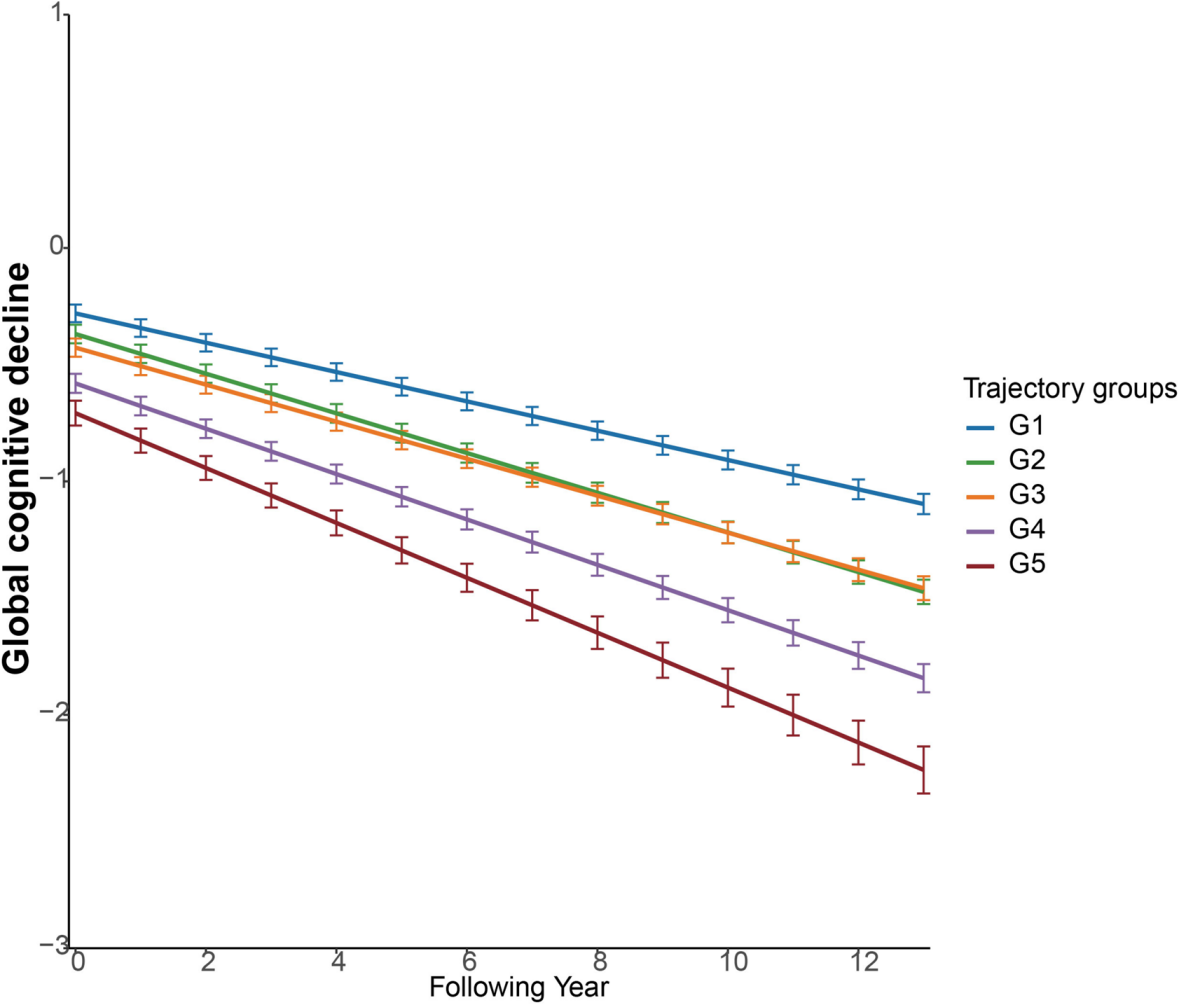
Trajectories of cognitive decline by multitrajectories of frailty and depression using multivariate mixed‐effects linear regression. G1: stable robust and nondepressed; G2: worsening prefrailty without depression; G3: stable prefrailty with escalating depressive symptoms; G4: increasing frailty alongside worsening depressive symptoms; G5: high and escalating frailty with persistent depression. The model was adjusted for baseline age, sex, race, marital status, educational level, wealth (in tertile), living alone, married status, smoking status and drinking once or more per week.

Table 2 shows details of results and Figure 2 presents the cognitive trajectories grouped by frailty and depression. Compared with G1, participants in G2, G3, G4 and G5 experienced an accelerated rate of cognitive decline. Specifically, the additional declines in cognitive function were −0.022 SD/year (95% CI: −0.030 to −0.015, *p* < 0.001), −0.016 SD/year (95% CI: −0.024 to −0.009, *p* < 0.001), −0.034 SD/year (95% CI: −0.043 to −0.025, *p* < 0.001) and −0.055 SD/year (95% CI: −0.071 to −0.039, *p* < 0.001), respectively.

**TABLE 2:** Multivariate mixed‐effects linear regression analysis of multitrajectories of frailty and depression with cognitive decline.

	Model 1		Model 2	
	*β* (95% CI)	*p*	*β* (95% CI)	*p*
Time, years	−0.063 (−0.067, −0.059)	< 0.001	−0.063 (−0.067, −0.059)	< 0.001
Multitrajectories of frailty and depression				
G1	Reference		Reference	
G2	−0.205 (−0.258, −0.153)	< 0.001	−0.088 (−0.138, −0.039)	< 0.001
G3	−0.315 (−0.369, −0.261)	< 0.001	−0.147 (−0.199, −0.095)	< 0.001
G4	−0.576 (−0.636, −0.516)	< 0.001	−0.300 (−0.359, −0.241)	< 0.001
G5	−0.840 (−0.935, −0.744)	< 0.001	−0.427 (−0.521, −0.334)	< 0.001
Multitrajectories of frailty and depression × time				
G1 × time	Reference		Reference	
G2 × time	−0.023 (−0.030, −0.015)	< 0.001	−0.022 (−0.030, −0.015)	< 0.001
G3 × time	−0.016 (−0.023, −0.009)	< 0.001	−0.016 (−0.024, −0.009)	< 0.001
G4 × time	−0.034 (−0.043, −0.025)	< 0.001	−0.034 (−0.043, −0.025)	< 0.001
G5 × time	−0.055 (−0.070, −0.039)	< 0.001	−0.055 (−0.071, −0.039)	< 0.001

*Note:* Model 1 was adjusted for baseline age and sex. Model 2 was adjusted for baseline age, sex, race, marital status, educational level, wealth (in tertile), living alone, married status, smoking status and drinking once or more per week.

Abbreviations: G1: stable robust and nondepressed; G2: worsening prefrailty without depression; G3: stable prefrailty with escalating depressive symptoms; G4: increasing frailty alongside worsening depressive symptoms; G5: high and escalating frailty with persistent depression.

### 3.3 Multitrajectories of Frailty and Depression With Dementia

The association of trajectory groups and dementia risk are shown in Table 3. There was an increased dementia risk in G2 (HR = 1.26, 95% CI: 1.08–1.48), G3 (HR = 1.54, 95% CI: 1.31–1.80), G4 (HR = 1.81, 95% CI: 1.54–2.14) and G5 (HR = 1.86, 95% CI: 1.48–2.33), compared with G1. The details are shown in Table 3.

**TABLE 3:** HR (95% CI) from the Cox proportional hazards models of dementia for multitrajectories of frailty and depression.

	Trajectories of frailty and depression				
	G1	G2	G3	G4	G5
No. of participants	3463	1801	1617	1285	434
Cases/person‐years	351/32704	310/14357	317/13923	318/8692	124/2539
Model 1	Reference	1.59 (1.36, 1.86)	2.08 (1.78, 2.42)	2.82 (2.41, 3.30)	3.91 (3.17, 4.83)
Model 2	Reference	1.26 (1.08, 1.48)	1.54 (1.31, 1.80)	1.81 (1.54, 2.14)	1.86 (1.48, 2.33)

*Note:* Model 1 was adjusted for baseline age and sex. Model 2 was adjusted for baseline age, sex, race, marital status, educational level, wealth (in tertile), living alone, married status, smoking status and drinking once or more per week.

Abbreviations: G1: stable robust and nondepressed; G2: worsening prefrailty without depression; G3: stable prefrailty with escalating depressive symptoms; G4: increasing frailty alongside worsening depressive symptoms; G5: high and escalating frailty with persistent depression.

### 3.4 Frailty and Depression Associated With Subdomains of Cognitive Decline

**FIGURE 3:**
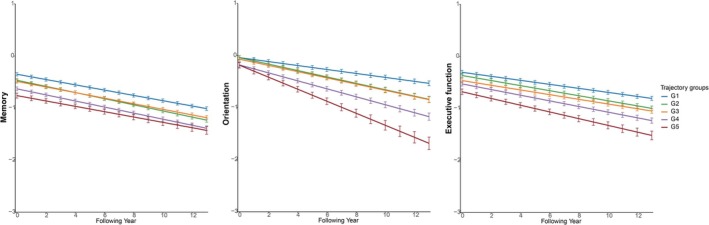
Trajectories of subdomains of cognitive function by multitrajectories of frailty and depression. G1: stable robust and nondepressed; G2: worsening prefrailty without depression; G3: stable prefrailty with escalating depressive symptoms; G4: increasing frailty alongside worsening depressive symptoms; G5: high and escalating frailty with persistent depression. The model was adjusted for baseline age, sex, race, marital status, educational level, wealth (in tertile), living alone, married status, smoking status and drinking once or more per week.

Table 4 shows details of the results, and Figure 3 presents trajectories of subdomains of cognitive decline by frailty and depression, respectively. Compared with G1, the decline in orientation function was more pronounced across all other groups. G4 and G5 are associated with reduced orientation (*β* = −0.038 and −0.078 SD/year, respectively) and executive function (*β* = −0.016 and −0.026 SD/year, respectively). Interestingly, G3 was associated with decreased orientation function (*β* = −0.022 SD/year; 95% CI: −0.031, −0.012, *p* < 0.001) and executive function −0.006 (*β* = −0.012, −0.001 SD/year; 95% CI: −0.012, −0.001, *p* = 0.027); G2 showed a slight but statistically significant acceleration of decline in memory (*β* = −0.008 SD/year), orientation (*β* = −0.024 SD/year) and executive function (*β* = −0.010 SD/year).

**TABLE 4:** Multivariate mixed‐effects linear regression analysis of multitrajectories of frailty and depression with subdomains of cognitive function.

	Memory		Orientation		Executive function	
*β* (95% CI)		*p*	*β* (95% CI)	P	*β* (95% CI)	*p*
Time, years	−0.051 (−0.054, −0.048)	< 0.001	−0.038 (−0.043, −0.033)	< 0.001	−0.039 (−0.042, −0.035)	< 0.001
Multitrajectories of frailty and depression						
G1	Reference		Reference		Reference	
G2	−0.116 (−0.161, −0.071)	< 0.001	−0.002 (−0.055, 0.051)	0.943	−0.061 (−0.110, −0.012)	0.014
G3	−0.138 (−0.184, −0.091)	< 0.001	−0.030 (−0.086, 0.026)	0.292	−0.159 (−0.209, −0.108)	< 0.001
G4	−0.280 (−0.334, −0.227)	< 0.001	−0.140 (−0.203, −0.076)	< 0.001	−0.223 (−0.281, −0.165)	< 0.001
G5	−0.410 (−0.495, −0.326)	< 0.001	−0.144 (−0.245, −0.043)	0.005	−0.373 (−0.465, −0.282)	< 0.001
Multitrajectories of frailty and depression × time						
G1 × time	Reference		Reference		Reference	
G2 × time	−0.008 (−0.014, −0.002)	0.005	−0.024 (−0.033, −0.014)	< 0.001	−0.010 (−0.016, −0.004)	0.001
G3 × time	−0.003 (−0.008, 0.003)	0.318	−0.022 (−0.031, −0.012)	< 0.001	−0.006 (−0.012, −0.001)	0.027
G4 × time	−0.007 (−0.014, −0.001)	0.035	−0.038 (−0.050, −0.027)	< 0.001	−0.016 (−0.023, −0.008)	< 0.001
G5 × time	−0.001 (−0.013, 0.012)	0.926	−0.078 (−0.098, −0.057)	< 0.001	−0.026 (−0.039, −0.013)	< 0.001

*Note:* The model was adjusted for baseline age, sex, race, marital status, educational level, wealth (in tertile), living alone, married status, smoking status and drinking once or more per week.

Abbreviations: G1: stable robust and nondepressed; G2: worsening prefrailty without depression; G3: stable prefrailty with escalating depressive symptoms; G4: increasing frailty alongside worsening depressive symptoms; G5: high and escalating frailty with persistent depression.

### 3.5 Sensitivity Analysis

We conducted a sensitivity analysis based on the composite cognitive scores derived from the summation of three domain‐specific measures and the results remained stable (Supplement table 6).

## 4 Discussion

This study characterized the trajectories of frailty and depression over mid‐ and late‐life and their association to cognitive decline and dementia in a large sample cohort study. The results revealed that 40.6% of participants followed a pattern of stable robust and nondepressed (G1), whereas nearly a quarter (20.9%) experienced worsening prefrailty without depression (G2). Additionally, 18.8% of participants exhibited a trajectory of stable prefrailty with escalating depressive symptoms(G3), 14.9% showed increasing frailty alongside worsening depressive symptoms(G4), and 5.1% reported high and escalating frailty with persistent depression (G5). There was an increased dementia risk in G2, G3, G4 and G5 (HR = 1.26, 1.54, 1.81 and 1.86, respectively), compared with G1. Notably, Groups 2, 4 and 5 showed declines in orientation and executive function subdomains, whereas Group 3's decline was limited to the orientation subdomain.

Previous studies have found that frailty and depression are risk factors for cognitive decline [2, 16], but most studies focused on a single time point or did not consider the combined effects of the two. Based on this, this study explored the impact of long‐term codevelopment of frailty and depression on cognitive decline and dementia.

This study found that high frailty and persistent depression were strongly associated with cognitive decline and dementia; the finding is consistent with previous research. The relationship between depression and cognitive decline has been well documented. Several longitudinal studies also support a significant association between persistent depression and cognitive decline [28, 29]. Moreover, existing literature suggests several potential mechanisms by which depression affects cognition. For instance, depression can lead to unhealthy lifestyle changes, such as poor diet and lack of physical activity, which are known to increase the risk of cognitive decline [30, 31, 32]. At the same time, the results of multiple longitudinal studies indicate that the early onset of depression, as well as the duration and frequency of depression, are associated with a twofold to fourfold increased risk of dementia [33, 34, 35]. Possible mechanisms associated with depression and dementia include inflammatory responses, vascular disease and hippocampal atrophy [10].

Research has shown a robust link association frailty with the deterioration of cognitive function and dementia. Several cross‐sectional studies indicate that frailty is linked to a higher risk of mild cognitive impairment in rural older adults [36], with greater cognitive impairment in frail individuals compared with those with prefrailty or robust health [37, 38]. Furthermore, evidence suggests that frailty may exacerbate the cognitive decline by augmenting the levels of chronic inflammation, thereby impairing neurological function [39, 40].

It is noteworthy that individuals with high and escalating frailty often experience persistent depression. Moreover, this group shows the most rapid cognitive decline and the highest risk of dementia over time. This may be attributed to bidirectional relationship between frailty and depression. Depression and frailty share common risk factors and pathophysiological pathways, such as chronic inflammation, oxidative stress, mitochondrial dysfunction and hypothalamic–adrenal axis dysregulation, which may contribute to their combined effects [15]. Depression may exacerbate frailty, and in turn, frailty may intensify depression, resulting in a combined effect on cognitive decline. This combined effect could be more pronounced than the impacts of either condition in isolation, thereby potentially accelerating the progression of cognitive deterioration and even causing dementia. These mechanisms may go part of to explain the combined effects of frailty and depressive trajectories on cognitive decline and an increased risk of dementia.

Lastly, our findings indicate that severe frailty and depression are particularly linked to declines in orientation abilities. The more significant impact on this subdomain might be attributed to its reliance on complex cognitive processes that are sensitive to both depression and frailty. This needs to be further explored in future studies.

Our study's findings indicate that integrating sustained screening for depression and frailty into strategies to prevent cognitive decline and dementia might improve preventative efficacy. Moreover, enhancing health education for middle‐aged and older adults to improve understanding of frailty and depressive symptoms is essential for achieving preventative objectives.

This study is a longitudinal analysis based on an HRS cohort, which is somewhat representative and allows for the exclusion of reverse causality. However, several limitations in this study can be observed. First, the assessment of frailty and depression relied on self‐reported data, which may introduce classification bias. Second, the assessment of trajectories required data from six waves, which could potentially result in healthy survivor bias. Finally, despite adjusting for a variety of covariates, it is possible that residual confounders remain, which may affect the accuracy of the study results.

## 5 Conclusion

The study examined the multitrajectories of frailty and depression and their joint impact on cognitive decline and dementia. Both worsening frailty and depression accelerate cognitive decline and dementia, with more pronounced effects for those experiencing higher levels of frailty and depression. This study might provide a new preventive perspective on cognitive decline and dementia by reducing levels of frailty or depression.

## Ethics Statement

The HRS was approved by the University of Michigan Institutional Review Board and the National Institute on Aging (HUM00061128), and written informed consent was obtained from all participants.

## Conflicts of Interest

The authors declare no conflicts of interest.

## Supporting information


**Data S1** Supplementary Information.

**Data S2** Supplementary Information.

## Data Availability

This study utilized publicly available data from the Health and Retirement Study. This data can be accessed using the following link: https://hrsdata.isr.umich.edu/data‐products/rand.
