# Supplementary material for: Multitrajectories of Frailty and Depression With Cognitive Function: Findings From the Health and Retirement Longitudinal Study
Source: J Cachexia Sarcopenia Muscle. 2025 Apr 6;16(2):e13795. doi: 10.1002/jcsm.13795 (PMC11972689; doi:10.1002/jcsm.13795)
Supplement: Supplementary file 1 — Data S1 Supplementary Information. [file JCSM-16-e13795-s002.docx]

Supplemental methods

**Step 1:** Initial screening of the number of trajectory groups. Group trajectory models with different numbers of trajectory groups (2~6) were constructed and cubic terms were fitted to the trajectory shape of each model. Censored normal models form was used, considering the continuous outcome of frailty index and CES-D score. We identified 5 trajectory groups as preliminary models (see Supplemental Table 1).

**Step 2:** Next, we determined the shape of each trajectory group. We started with high-order polynomial fittings for the subgroups within each model. If the higher-order terms were not significant, we proceeded with reduced-order fittings. Ultimately, we selected groups with model parameters **22133 - 33332 (BIC: -126461.01; AvepP, 0.83-0.96; Entropy: 0.895)**. The detailed information of Group trajectory model parameter estimates can be observed in Supplemental Tables 2-4.

The selection criteria included: 1) BIC of different models, the closer the negative value is to zero, the better the ﬁt; 2) adequate numbers of participants per group; 3) distinct trajectories (nonoverlapping CIs); 4) narrow CIs; 5) Proportion of each trajectory group greater than 5%; 6) average posterior probabilities of group membership >0.70; 7) Relative entropy (Ek) greater than 0.80; 8) odds of correct classification based on posterior probabilities of group membership, ensuring they exceeded 5. 9) the close correspondence between the estimated probabilities for each group and the proportion of participants classified into those groups using the maximum posterior probability assignment rule.
