# Supplementary material for: Multitrajectories of Frailty and Depression With Cognitive Function: Findings From the Health and Retirement Longitudinal Study
Source: J Cachexia Sarcopenia Muscle. 2025 Apr 6;16(2):e13795. doi: 10.1002/jcsm.13795 (PMC11972689; doi:10.1002/jcsm.13795)
Supplement: Supplementary file 2 — Data S2 Supplementary Information. [file JCSM-16-e13795-s001.docx]

**Supplemental Figure 1. Flowchart of selection.**


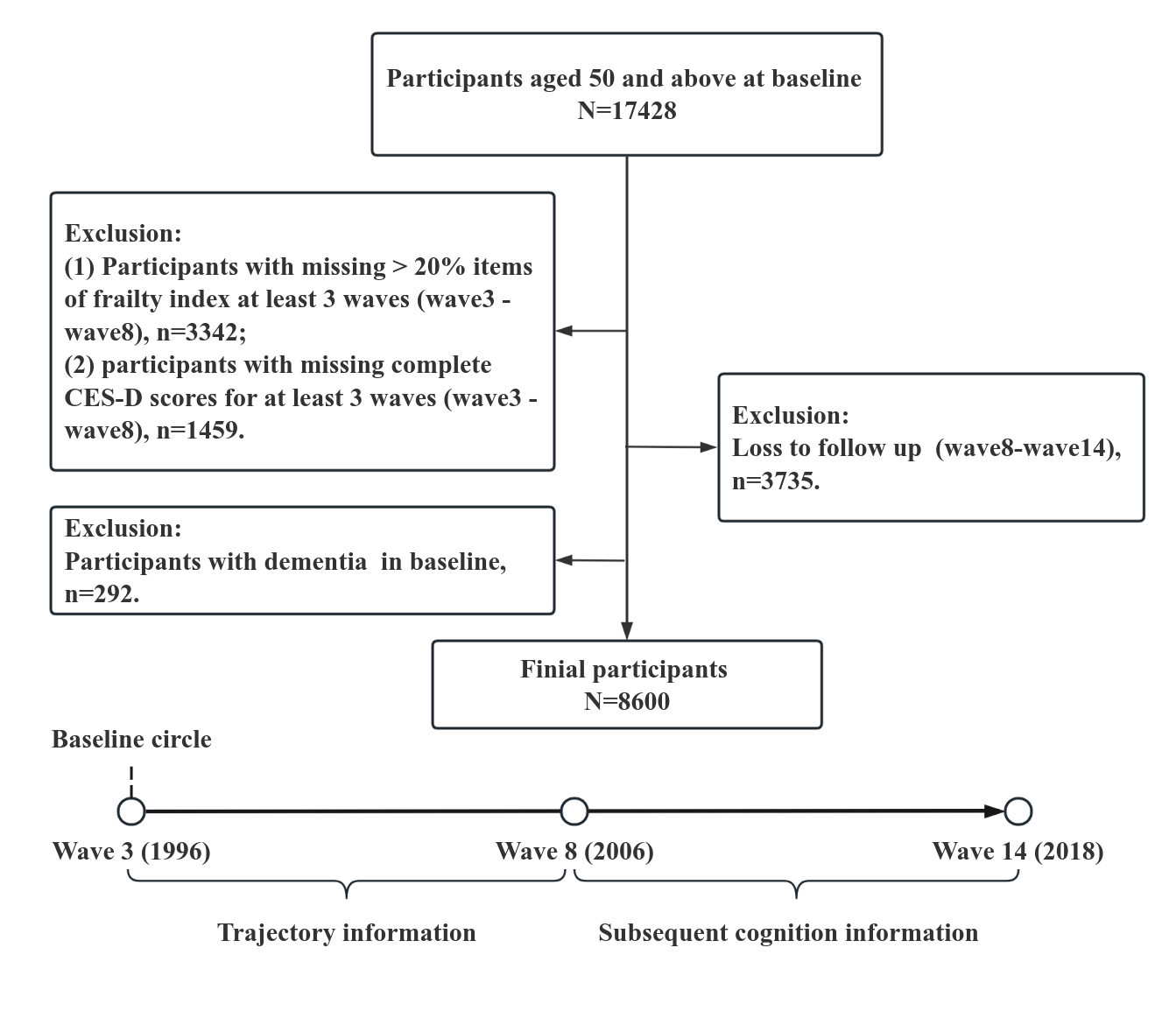


**Supplemental Table 1. Details definition of frailty.**

| **Health-related variables collected in waves 3-8** | | |
| --- | --- | --- |
| **Item** | **Variable/Code** | **Label** |
| **1** | **RwSHLT** | **rwshlt:ww self-report of health** |
| **2** | **RwHOSP** | **rwhosp:ww hospital stay, prv 2 yrs** |
| **3** | **RwNRSHOM** | **rwnrshom:ww nurs home stay, prv 2 yrs** |
| **4** | **RwNHMLIV** | **rwnhmliv:ww live in nurs home at iview** |
| **5** | **RwDOCTIM** | **rwdoctim:ww # doctor vists, prv 2 yrs** |
| **6** | **RwHOMCAR** | **rwhomcar:ww home hlth care, prv 2 yrs** |
| **7** | **RwOUTPT** | **rwoutpt:ww outpatient surgry, prv 2 yrs** |
| **8** | **RwSPCFAC** | **rwspcfac:ww spec hlth facilty, prv 2 yrs** |
| **9** | **RwHIBPE** | **rwhibpe:ww r ever had high blood pressure** |
| **10** | **RwDIABE** | **rwdiabe:ww r ever had diabetes** |
| **11** | **RwCANCRE** | **rwcancre:ww r ever had cancer** |
| **12** | **RwLUNGE** | **rwlunge:ww r ever had lung disease** |
| **13** | **RwHEARTE** | **rwhearte:ww r ever had heart problems** |
| **14** | **RwSTROKE** | **rwstroke:ww r ever had stroke** |
| **15** | **RwARTHRE** | **rwarthre:ww r ever had arthritis** |
| **16** | **RwWALKR** | **rwwalkr:ww r diff-walk across room** |
| **17** | **RwWALKRH** | **rwwalkrh:ww r gets help-walk across room** |
| **18** | **RwWALKRE** | **rwwalkre:ww r eqp-walk across room** |
| **19** | **RwDRESS** | **rwdress:ww r diff-dressing** |
| **20** | **RwDRESSH** | **rwdressh:ww r gets help-dressing** |
| **21** | **RwBATH** | **rwbath:ww r diff-bathing or showerng** |
| **22** | **RwBATHH** | **rwbathh:ww r gets help-bathing, showerng** |
| **23** | **RwEAT** | **rweat:ww r diff-eating** |
| **24** | **RwEATH** | **rweath:ww r gets help-eating** |
| **25** | **RwBED** | **rwbed:ww r diff-get in/out of bed** |
| **26** | **RwBEDH** | **rwbedh:ww r gets help-get in/out of bed** |
| **27** | **RwBEDE** | **rwbede:ww r use eqp-get in/out of bed** |
| **28** | **RwTOILT** | **rwtoilt:ww r diff-using the toilet** |
| **29** | **RwTOILTH** | **rwtoilth:ww r gets help-using the toilet** |
| **30** | **RwPHONE** | **rwphone:ww r diff-use telephone** |
| **31** | **RwMONEY** | **rwmoney:ww r diff-managing money** |
| **32** | **RwSHOP** | **rwshop:ww r diff-shop for groceries** |
| **33** | **RwWALKS** | **rwwalks:ww r diff-walk sev blocks** |
| **34** | **RwWALK1** | **rwwalk1:ww r diff-walk one block** |
| **35** | **RwCHAIR** | **rwchair:ww r diff-get up fr chair** |
| **36** | **RwCLIMS** | **rwclims:ww r diff-climb sev flt stair** |
| **37** | **RwCLIM1** | **rwclim1:ww r diff-climb one flt stair** |
| **38** | **RwSTOOP** | **rwstoop:ww r diff-stoop/kneel/crouch** |
| **39** | **RwLIFT** | **rwlift:ww r diff-lift/carry 10lbs** |
| **40** | **RwDIME** | **rwdime:ww r diff-pick up a dime** |
| **41** | **RwARMS** | **rwarms:ww r diff-reach/extnd arms up** |
| **42** | **RwPUSH** | **rwpush:ww r diff-push/pull large obj** |
| **43** | **RwSIGHT** | **rwsight:ww r self-rated eyesight** |
| **44** | **RwDSIGHT** | **rwdsight:ww r self-rated distance eyesight** |
| **45** | **RwNSIGHT** | **rwnsight:ww r self-rated near eyesight** |
| **46** | **RwHEARING** | **rwhearing:ww r self-rated hearing** |
| **47** | **RwHEARAID** | **rwhearaid:ww r wears hearing aid** |
| **48** | **RwURINAI** | **rwurinai:ww r any urinary incontinence** |
| **49** | **RwURINAF** | **rwurinaf:ww r number days urinary incontinence** |
| **50** | **RwHRTATT** | **rwhrtatt:ww r reports heart attack since last wave** |
| **51** | **RwCONHRTF** | **rwconhrtf:ww r reports congestive heart failure since last w** |
| **52** | **RwRXHIBP** | **rwrxhibp:ww r takes meds for high blood pressure** |
| **53** | **RwRXDIABO** | **rwrxdiabo:ww r takes oral meds for diabetes** |
| **54** | **RwRXDIABI** | **rwrxdiabi:ww r takes insulin for diabetes** |
| **55** | **RwRXSTROK** | **rwrxstrok:ww r takes meds for stroke** |
| **56** | **RwRXLUNG** | **rwrxlung:ww r takes meds for lung condition** |
| **57** | **RwHRTSRG** | **rwhrtsrg:ww r had heart surgery since last wave** |
| **58** | **RwJOINTR** | **rwjointr:ww r had joint replaced since last wave** |
| **59** | **RwBMICAT** | **rwbmicat:ww r bmi categorization** |
| **60** | **RwLIMHWRK** | **rwlimhwrk:ww whether health limits r housework** |
| **61** | **RwLIMIMPAR** | **rwlimimpar:ww whether r limited in anyway due to impairment** |

**Supplemental Figure 2. Frequency distribution of frailty index:Wave 3 as an Example .**

**
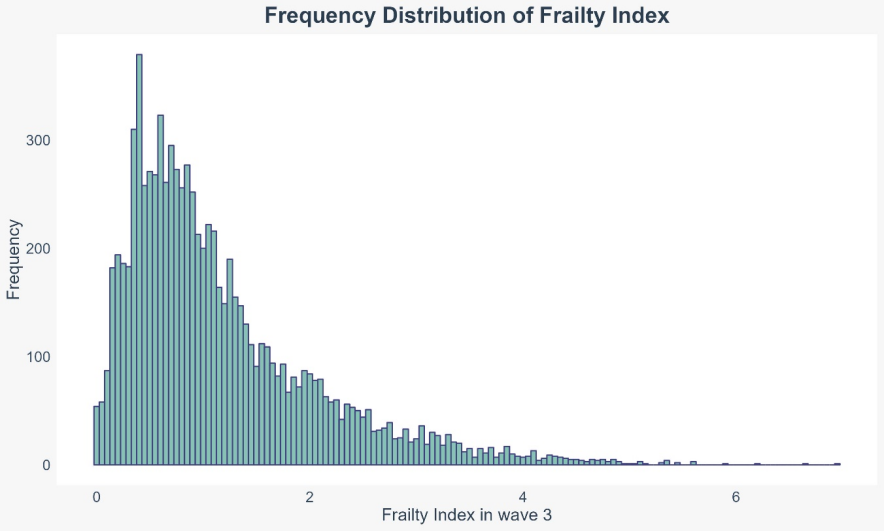
**

**Supplemental Figure 3. The relationship between frailty index and age stratified by Gender:Wave 3 as an Example .**

**
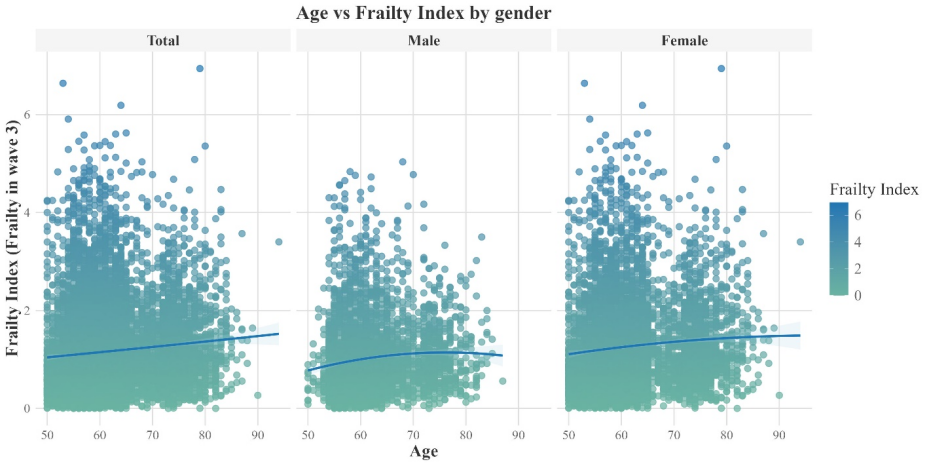
**

**Supplemental description. Description of frailty index: Wave 3 as an Example.**

**The frailty index showed a right-skewed distribution (Supplemental Figure 2) and a non-linear relationship with age (Supplemental Figure 3), with a significant Spearman correlation of 0.14 (P < 0.001). Females had significantly higher scores than males, with means of 1.27 (SD = 0.97) vs. 1.02 (SD = 0.78) and medians of 0.99 (IQR: 0.56-1.77) vs. 0.82 (IQR: 0.46-1.32), respectively.**

Supplemental Table 2. Model fit for trajectories of frailty and depression.

| Number of classes | Parameters of trajectory shape | Proportions per class% | AvepP | BIC | Entropy |
| --- | --- | --- | --- | --- | --- |
| 2 | 33-33 | 71.38-28.61 | 0.97-0.99 | -140560.51 | 0.944 |
| 3 | 333-333 | 50.52-14.22 | 0.94-0.97 | -133140.39 | 0.906 |
| 4 | 3333-3333 | 49.33-11.35 | 0.91-0.97 | -129262.48 | 0.901 |
| 5 | 33333-33333 | 40.39-7.26 | 0.90-0.96 | -126524.86 | 0.890 |
| 6 | 333333-333333 | 35.48-4.77 | 0.89-0.94 | -124478.47 | 0.886 |
| Best fit |  |  |  |  |  |
| 5 | 22133-33332 | 39.75-5.08 | 0.83-0.96 | -126461.01 | 0.895 |

Parameters of trajectory shape: Frailty-Depression; Trajectory shape:1 = linear, 2 = quadratic,3 = Cubic; AvepP: Average posterior probability; BIC = Bayesian information criterion.

Supplemental Table 3. Group trajectory model parameter estimates (based on model 22133-33332).

| Frailty - Depression | Group | Parameter | Estimate | Standard error | T | *P* |
| --- | --- | --- | --- | --- | --- | --- |
| Frailty | G1 | Intercept | 0.600 | 0.010 | 60.947 | < 0.001 |
|  |  | Linear | 0.010 | 0.004 | 2.376 | 0.018 |
|  |  | Quadratic | 0.003 | 0.001 | 6.984 | < 0.001 |
|  | G2 | Intercept | 1.338 | 0.016 | 84.077 | < 0.001 |
|  |  | Linear | 0.035 | 0.006 | 5.742 | < 0.001 |
|  | G3 | Intercept | 0.006 | 0.001 | 10.797 | < 0.001 |
|  |  | Linear | 0.88 | 0.013 | 66.313 | < 0.001 |
|  | G4 | Intercept | 0.053 | 0.002 | 27.379 | < 0.001 |
|  |  | Linear | 2.076 | 0.02 | 101.579 | < 0.001 |
|  |  | Quadratic | 0.125 | 0.016 | 7.986 | 0.001 |
|  |  | Cubic | -0.013 | 0.004 | -3.491 | < 0.001 |
|  | G5 | Intercept | 0.001 | 0.001 | 5.314 | < 0.001 |
|  |  | Linear | 3.331 | 0.032 | 104.923 | < 0.001 |
|  |  | Quadratic | 0.235 | 0.027 | 8.758 | < 0.001 |
|  |  | Cubic | -0.03 | 0.007 | -4.662 | < 0.001 |
| Depression | G1 | Intercept | 0.002 | 0.001 | 4.716 | < 0.001 |
|  |  | Linear | -1.11 | 0.034 | -32.403 | < 0.001 |
|  |  | Quadratic | 0.335 | 0.029 | 11.552 | < 0.001 |
|  |  | Cubic | -0.085 | 0.007 | -12.081 | < 0.001 |
|  | G2 | Intercept | 0.005 | 0.001 | 11.237 | < 0.001 |
|  |  | Linear | -0.576 | 0.04 | -14.561 | < 0.001 |
|  |  | Quadratic | 0.296 | 0.03 | 9.722 | < 0.001 |
|  |  | Cubic | -0.068 | 0.007 | -9.811 | < 0.001 |
|  | G3 | Intercept | 0.004 | 0.001 | 10.029 | < 0.001 |
|  |  | Linear | 0.663 | 0.022 | 29.601 | < 0.001 |
|  |  | Quadratic | 0.156 | 0.017 | 9.154 | < 0.001 |
|  |  | Cubic | -0.03 | 0.004 | -7.501 | < 0.001 |
|  | G4 | Intercept | 0.002 | 0.001 | 6.369 | < 0.001 |
|  |  | Linear | 0.846 | 0.022 | 38.834 | < 0.001 |
|  |  | Quadratic | 0.105 | 0.016 | 6.390 | < 0.001 |
|  |  | Cubic | -0.017 | 0.004 | -4.323 | 0.001 |
|  | G5 | Intercept | 0.001 | 0.001 | 3.471 | < 0.001 |
|  |  | Linear | 1.283 | 0.025 | 51.181 | 0.001 |
|  |  | Quadratic | 0.037 | 0.011 | 3.498 | 0.001 |

Abbreviations: G1: Stable robust, and non-depressed; G2: Worsening pre-frailty without depression; G3: Stable pre-frailty with escalating depressive symptoms; G4: Increasing frailty alongside worsening depressive symptoms; G5: High and escalating frailty with persistent depression.

Supplemental Table 4. Odds of correct classification for trajectories of frailty and depression.

| Trajectory group | OCC | OCCw |
| --- | --- | --- |
| G 1 | 28.78 | 29.41 |
| G 2 | 33.65 | 33.13 |
| G 3 | 44.37 | 43.89 |
| G 4 | 105.08 | 104.54 |
| G 5 | 535.97 | 532.49 |

Abbreviations: G1: Stable robust, and non-depressed; G2: Worsening pre-frailty without depression; G3: Stable pre-frailty with escalating depressive symptoms; G4: Increasing frailty alongside worsening depressive symptoms; G5: High and escalating frailty with persistent depression.

OCC: Odds of correct classification;

OCCw: Odds of correct classification using weighted posterior probabilities.

Supplemental Table 5. Frailty and depression trajectories groups estimated probability and the proportion of participants classified to each group according to the maximum posterior probability assignment rule.

| Trajectory group | Estimated group probability, based on the sums of posterior probabilities (%) | Proportion assigned to group according to the maximum posterior probability assignment rule (%) |
| --- | --- | --- |
| G 1 | 39.75 | 40.27 |
| G 2 | 21.20 | 20.94 |
| G 3 | 18.97 | 18.80 |
| G 4 | 15.01 | 14.94 |
| G 5 | 5.08 | 5.05 |

Abbreviations: G1: Stable robust, and non-depressed; G2: Worsening pre-frailty without depression; G3: Stable pre-frailty with escalating depressive symptoms; G4: Increasing frailty alongside worsening depressive symptoms; G5: High and escalating frailty with persistent depression.

Supplemental Table 6. Multivariate mixed-effects linear regression analysis of multi-trajectories of frailty and depression with cognitive decline based on the original cognitive scores .

|  | Model 1 | |  | Model 2 | |
| --- | --- | --- | --- | --- | --- |
|  | β (95% CI) | *P* |  | β (95% CI) | *P* |
| Time, years | -0.095 (-0.100, -0.09) | <0.001 |  | -0.095 (-0.100, -0.09) | <0.001 |
| Multi-trajectories of frailty and depression | | | | | |
| G1 | Reference |  |  | Reference |  |
| G2 | -0.359 (-0.438, -0.280) | <0.001 |  | -0.168 (-0.241, -0.094) | <0.001 |
| G3 | -0.536 (-0.617, -0.455) | <0.001 |  | -0.257 (-0.334, -0.181) | <0.001 |
| G4 | -0.933 (-1.023, -0.842) | <0.001 |  | -0.489 (-0.577, -0.402) | <0.001 |
| G5 | -1.400 (-1.543, -1.257) | <0.001 |  | -0.738 (-0.876, -0.601) | <0.001 |
| Multi-trajectories of frailty and depression × time | | | | | |
| G1 × time | Reference |  |  | Reference |  |
| G2 × time | -0.022 (-0.031, -0.013) | <0.001 |  | -0.021 (-0.031, -0.012) | <0.001 |
| G3 × time | -0.014 (-0.023, -0.005) | <0.001 |  | -0.014 (-0.023, -0.004) | 0.004 |
| G4 × time | -0.030 (-0.041, -0.018) | <0.001 |  | -0.030 (-0.042, -0.019) | <0.001 |
| G5 × time | -0.038 (-0.058, -0.018) | <0.001 |  | -0.036 (-0.057, -0.016) | 0.001 |

Abbreviations: G1: Stable robust, and non-depressed; G2: Worsening pre-frailty without depression; G3: Stable pre-frailty with escalating depressive symptoms; G4: Increasing frailty alongside worsening depressive symptoms; G5: High and escalating frailty with persistent depression.

Model 1 was adjusted for baseline age, sex.

Model 2 was adjusted for baseline age, sex, race, marital status, educational level, wealth (in tertile), living alone, married status, smoking status, and drinking once or more per week.
